# Variability in Positions and Factors Contributing to Surgical Difficulty of Impacted Third Molars

**DOI:** 10.1055/s-0044-1788796

**Published:** 2024-09-27

**Authors:** Endang Sjamsudin, Anggun Rafisa, Nuroh Najmi

**Affiliations:** 1 Department of Oral and Maxillofacial Surgery, Faculty of Dentistry, Universitas Padjadjaran, Indonesia; 2 Department of Oral Biology, Faculty of Dentistry, Universitas Padjadjaran, Indonesia

**Keywords:** impaction, third molar, angulation, occlusal plane, surgery time, surgery technique

## Abstract

**Objectives** This study aimed to provide valuable insights into the variability of third molar positions and factors influencing their surgical time and technique.

**Materials and Methods** This cross-sectional study included a total of 48 eligible participants, aged 18 to 45 years, diagnosed with impacted teeth, and who had undergone surgery. Exclusion criteria comprised the absence of the second molar, the presence of systemic diseases, a history of radiation therapy, and pregnancy or lactation. Participants completed a questionnaire covering demographic data, physical metrics, and information on systemic conditions and disorders. Preoperative assessments included vital sign measurements. Panoramic imaging was employed to evaluate the third molar distance to the occlusal plane, degrees of angulation, eruption space, and distance to the alveolar inferior canal. Surgical difficulty in this study was measured by two outcomes: surgical time and technique.

**Statistical Analysis** To assess differences in impacted third molar positions among groups, Independent samples *t* -test and One-way analysis of variance were used for normally distributed data without outliers; otherwise, the Mann–Whitney U test and Kruskal–Wallis H test were utilized. The Spearman's rank correlation was utilized to explore relationships between vital signs, impacted third molar positions, surgical time, and surgical technique.

**Results** There were no significant differences in third molar positions between age and body mass index groups. Significant differences in distance to the occlusal plane were observed between third molars in quadrants 2 and 3 ( *p* = 0.002) and quadrants 2 and 4 ( *p* = 0.005). A significant difference in eruption space was found between sexes ( *p* = 0.016). A significant negative correlation was discovered between surgical time and respiration rate per minute ( *p* = 0.028).

**Conclusion** This study found that males have greater third molar eruption space than females, and maxillary third molars have a greater distance to the occlusal plane compared with mandibular third molars. The importance of vital signs as contributing factors to surgical difficulty is highlighted, emphasizing their relevance in clinical practice.

## Introduction

Impacted third molars pose a recurrent issue in oral and maxillofacial surgery. These molars, also known as wisdom teeth, present distinctive challenges compared with other teeth due to their heightened susceptibility to disease, nonfunctional nature, and unique anatomical characteristics. 1 Third molars encounter limitations in physiological space and maintenance as the last teeth erupt in the dental arch. This often results in compromised soft tissue support, leading to bacterial accumulation and inflammation. In cases where disease is visible, removing third molars is a practical and cost-effective way to improve oral health. 1 2

The management of impacted third molars requires a comprehensive understanding of their diverse positions and associated factors. Some studies have suggested that demographic factors such as sex and age may potentially influence the variability of third molar positions. 3 4 5 Additionally, nutritional factors have been suggested to impact tooth eruption dynamics, with overweight individuals potentially experiencing the earlier eruption of the third molar 6 and exhibiting larger mandibular and maxillary dimensions. 7 However, conflicting results exist in other studies, indicating that third molar positions did not differ statistically between sex groups 4 8 and that body mass index (BMI) did not predict the occurrence of tooth impaction. 9

Numerous studies have explored variables influencing the surgical difficulty of impacted third molar extraction, examining dental factors alone 10 11 or incorporating dental, physical, and demographic factors. 12 These investigations consistently emphasize the impact of third molar positions on surgical time and technique. However, to our knowledge, vital signs have not been investigated as potential factors affecting surgical difficulty. Vital signs offer an objective measure of critical physiological functions that are crucial for identifying and assessing effective responses to emergencies. 13 Changes in vital signs may be induced during dental extraction due to drugs, local anesthetics, previous dental experience, or anxiety. 14 15

This study aimed to contribute valuable insights into the variability in third molar positions and factors contributing to their surgical time and technique. Such knowledge is fundamental for developing treatment plans, enhancing surgical efficiency, and ensuring optimal patient outcomes in the field of oral and maxillofacial surgery.

## Materials and Methods

This cross-sectional study was conducted on patients seeking evaluation and surgical treatment for impacted third molars at the Department of Oral and Maxillofacial Surgery, RSGM Universitas Padjadjaran in 2023. The total sampling technique was employed, and a total of 48 eligible participants, aged 18 to 45 years, diagnosed with impacted teeth, and having undergone surgery for impacted third molars, were included after providing informed consent. Exclusion criteria consisted of the absence of the second molar, the presence of systemic diseases such as hypertension, diabetes mellitus, or kidney diseases, a history of radiation therapy, and pregnancy or lactation.

### Data Collection

Participants were administered a questionnaire addressing demographic data (age, sex, educational background, and occupation), physical metrics (weight and height), and information on systemic conditions and disorders. Preoperative assessments included vital sign measurements, encompassing pulse rate through radial artery palpation, blood pressure measured using the TensiOne 1A Onemed automated sphygmomanometer (PT. Jayamas Medica Industri Tbk, Sidoarjo, Indonesia), respiratory rate counted visually based on chest or abdomen movement per minute, and axillary temperature measured in degrees Celsius using the ThermoONE Alpha 1 Onemed digital thermometer (PT. Jayamas Medica Industri Tbk, Sidoarjo, Indonesia). Vital signs were measured twice before the surgical procedures, and the average of these two measurements was used for analysis.

### Radiographic Evaluation

Panoramic imaging was employed to evaluate the positions of impacted third molars. A single examiner, with over 2 years of experience as an oral and maxillofacial radiologist, assessed all panoramic images. The evaluation criteria included the distance of the impacted third molars to the occlusal plane, degrees of angulation (the inclination of the third molar to the long axis of the second molar), and eruption space (mandibular retromolar space and maxillary tuberosity space). Additionally, the distance from the apex of the third molar to the inferior alveolar canal and the distance from the second molar to the inferior alveolar canal were measured. The panoramic images were imported using CliniView Imaging 11.7, and linear measurements were conducted with ImageJ software, version 1.54.

### Surgical Difficulty Assessment

The surgical difficulty in this study was measured by two outcome variables, which were surgical time and surgical technique. The surgery time was calculated by measuring the time elapsed between incision and tissue suturing in minutes. The surgical technique was classified into three levels of difficulty: low difficulty (extraction performed solely with an elevator), moderate difficulty (ostectomy required), and high difficulty (both ostectomy and tooth sectioning performed). 12 Each level of difficulty was assigned a numerical value: low = 1, moderate = 2, and high = 3.

### Statistical Analysis

All data were analyzed using IBM SPSS Statistics, version 26.0. Intrarater reliability was assessed by repeating all linear measurements 1 week after the initial measurements and calculating the Intraclass Correlation Coefficients (ICCs) based on single measurements, absolute agreement, and a two-way mixed-effects model. Boxplots were used to identify potential outliers within variable groups, and the Shapiro–Wilk test was conducted to assess the normality of data distribution. Differences in impacted third molar positions among groups were analyzed using parametric tests (Independent samples *t* -test and One-way analysis of variance) if no outliers were found and data were normally distributed; otherwise, nonparametric tests (Mann–Whitney U test and Kruskal–Wallis H test) were applied. Additionally, Spearman's rank correlation was used to explore relationships between vital signs, impacted third molar positions, surgical time, and surgical technique. Statistical significance was set at *p* < 0.05.

### Ethics

All patients received comprehensive information regarding the study procedures and provided their informed consent by signing a consent statement. The study obtained ethical approval from the Research Ethics Committee of Universitas Padjadjaran Bandung (Approval Number 999/UN6.KEP/EC/2023).

## Results

The analysis of ICCs indicated high intrarater reliability for each variable studied: distance to the occlusal plane, ICC = 0.945 (95% CI, 0.879–0.972); impaction angle, ICC = 0.981 (95% CI, 0.967–0.990); eruption space, ICC = 0.847 (95% CI, 0.743–0.911); distance from third molar apex to the inferior alveolar canal, ICC = 0.928 (95% CI, 0.865–0.962); and distance from second molar apex to the inferior alveolar canal, ICC = 0.901 (95% CI, 0.815–0.948).

Table 1 presents the characteristics of participants in this study. The distribution across sex groups showed a higher number of females (62.5%). Age distribution indicated a higher number of individuals over 25 years old (60.4%). Additionally, a significant proportion of the study subjects (75.0%) exhibited normal nutritional status, with a BMI within the range of 18.5 to 24.9 kg/m ^2^ . The measurement of vital signs showed mean results within normal ranges. Third molar impaction was more prevalent in the mandible (77.1%) compared with the maxilla (22.9%). Based on quadrant location, the highest number of participants experienced impacted third molars in quadrant 4.

**Table 1:** Descriptive statistics of the study variables

Categorical variables		Frequency		Percentage (%)	
Study subjects' characteristics	Age (years)				
	< 25	19		39.6	
	≥ 25	29		60.4	
	Sex					
	Male	18		37.5	
	Female	30		62.5	
	BMI					
	< 18.5	4		8.3	
	18.5–24.9	36		75.0	
	≥ 25	8		16.7	
Impacted third molars' characteristics	Jaw location				
	Maxilla	11		22.9	
	Mandible	37		77.1	
	Quadrant location					
	1	4		8.3	
	2	7		14.6	
	3	14		29.2	
	4	23		47.9	
	Surgical technique					
	Low difficulty	11		22.9	
	Moderate difficulty	33		68.8	
	High difficulty	4		8.3	
Continuous variables		Mean	SD	Median	IQR
Study subjects' characteristics	Age (years)	28.04	6.54	26.00	10.50
	BMI	22.71	3.22	22.46	3.66
	Pulse rate per minute	87.08	11.63	88.00	16.00
	Systolic blood pressure (mm Hg)	114.04	12.47	114.50	13.50
	Diastolic blood pressure (mm Hg)	76.77	8.59	78.50	10.75
	Respiration rate per minute	20.00	0.36	20.00	0.00
	Body temperature (°C)	36.26	0.42	36.20	0.60
Impacted third molars' characteristics	Distance to the occlusal plane (mm)	4.41	3.02	3.50	3.03
	Impaction angle (degrees)	37.47	27.25	35.25	37.60
	Eruption space (mm)	10.38	3.456	10.72	3.89
	Distance from third molar apex to the inferior alveolar canal (mm)	2.28	2.00	1.89	2.57
	Distance from second molar apex to the inferior alveolar canal (mm)	2.16	1.96	1.90	3.00
	Surgical time (minutes)	26.73	13.38	26.50	12.75

Abbreviations: BMI, body mass index; IQR, interquartile range; SD, standard deviation.

The analysis of the positions of impacted third molars between groups ( Table 2 ) showed a statistically significant difference in the distance to the occlusal plane between third molars in different jaws ( *p* = 0.000) and quadrant locations ( *p* = 0.001). Post hoc analysis using Dunn's procedure with a Bonferroni correction revealed statistically significant differences in the distance to the occlusal plane between third molars in quadrant 2 and quadrant 3 ( *p* = 0.002), as well as between quadrant 2 and quadrant 4 ( *p* = 0.005). Furthermore, there was a significant difference in eruption space between sex groups ( *p* = 0.016). Correlation analysis for surgical variables ( Table 3 ) revealed a statistically significant negative correlation between surgical time and respiration rate per minute ( *p* = 0.028).

**Table 2:** The comparison of the impacted third molar positions between groups based on study participants' characteristics and impacted third molar locations

Variables		Distance to the occlusal plane (mm)	*p* -Value	Impaction angle (degrees)	*p* -Value	Eruption space (mm)	*p* -Value	Distance from third molar apex to the inferior alveolar canal (mm)	*p* -Value	Distance from second molar apex to the inferior alveolar canal (mm)	*p* -Value
Study subjects' characteristics	Age (years)											
	< 25	3.96 (2.97)	0.480 a	38.66 (35.69)	0.422 a	9.20 ± 4.52	0.095 b	1.80 (2.41)	0.913 a	1.42 (3.06)	0.278 a
	≥25	3.12 (2.84)		33.29 (40.90)		11.16 ± 2.32		1.90 (3.33)		1.94 (2.82)	
	Sex										
	Male	3.81 (2.94)	0.412 a	35.08 (50.09)	0.717 a	11.73 ± 2.10	0.016 b c	1.84 (2.15)	0.226 a	3.04 (3.70)	0.314 a
	Female	3.11 (13.15)		35.25 (35.49)		9.57 ± 3.87		1.91 (2.73)		1.75 (2.39)	
	BMI										
	< 18.5	4.20 (4.37)	0.855 c	29.03 ± 22.16	0.373 d	12.79 (6.58)	0.245 e	0.00 (0.00)	0.115 e	2.36 (0.00)	0.022 c e
	18.5–24.9	3.65 (3.02)		40.68 ± 27.02		10.17 (4.06)		1.91 (2.70)		2.35 (2.31)	
	≥ 25	3.40 (4.86)		27.22 ± 30.21		11.23 (4.37)		1.33 (3.00)		0.14 (0.68)	
Impacted third molars' locations	Jaw										
	Maxilla	7.52 (5.8)	0.000 a c	20.85 (41.46)	0.071 a	9.08 (5.52)	0.425 a	–			
	Mandible	3.07 (2.22)		37.80 (38.16)		11.06 (3.52)		1.89 (2.57)	–	1.90 (2.99)	–
	Quadrant										
	1	6.80 (4.36)	0.001 c e	13.30 (37.74)	0.062 e	12.58 (9.52)	0.339 e	–			
	2	11.45 (7.51)		20.85 (84.92)		9.08 (4.16)					
	3	2.98 (2.22)		54.12 (54.40)		9.99 (3.72)		1.20 (3.02)	0.510 a	1.41 (2.62)	0.226 a
	4	3.12 (2.15)		35.01 (30.45)		11.80 (3.66)		1.93 (2.18)		2.34 (3.18)	

Abbreviation: BMI, body mass index.

Parametric tests are represented by mean ± standard deviation, while nonparametric tests are represented by median (interquartile range).

This table legend should be removed as the table format has been adjusted to align with the journal's format.

a Mann–Whitney U test.

b Independent samples *t* -test.

c Statistically significant difference ( *p* < 0.05).

d One-way analysis of variance.

e Kruskal–Wallis H test.

**Table 3:** The correlation between vital signs, impacted third molar positions, surgical time and surgical technique

Variables	Surgical time		Surgical technique	
	* r _s_*	*p* -Value	* r _s_*	*p* -Value
Vital signs				
Pulse rate per minute	0.105	0.477	− 0.112	0.449
Systolic blood pressure (mm Hg)	0.218	0.137	− 0.025	0.865
Diastolic blood pressure (mm Hg)	0.062	0.678	− 0.096	0.514
Respiration rate per minute	− 0.318	0.028 a	− 0.138	0.351
Body temperature (°C)	− 0.180	0.222	0.160	0.277
Impacted third molar positions				
Distance to the occlusal plane (mm)	− 0.260	0.074	0.183	0.212
Impaction angle (degrees)	0.259	0.075	0.140	0.342
Eruption space (mm)	− 0.104	0.482	− 0.025	0.866
Distance from third molar apex to the inferior alveolar canal (mm)	0.125	0.461	− 0.082	0.630
Distance from second molar apex to the inferior alveolar canal (mm)	− 0.121	0.476	0.007	0.969

Abbreviation: *r _s_* = Spearman's rank correlation coefficient.

a Statistically significant correlation ( *p* < 0.05).

## Discussion

This study investigated the variability in the positions of impacted third molars using linear measurements from panoramic radiographs rather than using classification or index that have been developed before which have been studied in many previous studies. 16 17 18 19 In this study, a statistically significant difference was found in the eruption space between the sex group and distance to the occlusal plane between third molars in different jaw and quadrant locations. However, no statistically significant differences in third molars' positions were observed between groups based on age and BMI.

This study found that the male subjects with impacted third molar had greater eruption space than females. This finding is supported by the previous studies by Al-Gunaid 3 and Yilmaz et al, 20 which found greater retromolar space in males and a study by Manzanera et al, 21 which found larger tuberosity space in males. However, a study reported by Hattab and Alhaija, 22 which only included impacted third molar with mesioangular angulation found no significant difference in retromolar space between males and females who had impacted third molar, but found a significant difference of retromolar space between males and females with erupted third molar. Other studies by Guo et al 23 also discovered no difference in retromolar space between sexes.

The different eruption spaces between sexes in the present data may be explained by different amount of sizes and shapes of facial growth in males and females. Compared with males, females have a smaller amount and relatively more vertical direction. 24 The trend of growth between sexes is also different. Females grow in more amount earlier but the growth ceases at a younger age than males. Females have larger retromolar spaces around puberty, but postpuberty, males show a larger magnitude of growth going forward. 25

Data in this study discovered that maxillary third molars, particularly the one in quadrant 2, were found to have a significantly larger distance to the occlusal plane compared with mandibular third molars. Several studies exploring the depth of the third molar predominantly used the Pell and Gregory classification, of which the results are still in dispute. Primo et al 26 and Khouri et al 27 reported that maxillary third molars have a deeper position (level C) relative to the second molar occlusal plane than the mandibular third molar (level B). A study by Yilmaz et al 20 revealed that the most prevalent position of the maxillary third molar is level B, and the most prevalent position of the mandibular third molar is level C. Kaomongkolgit and Tantanapornkul 16 identified level B as the most prevalent occlusal plane level for third molars in the maxillary and mandible.

The absence of a significant difference in impacted third molar positions between age groups contradicts findings from a previous study by Ryalat et al, 4 which found a decrease in distance to the occlusal plane and an increase in retromolar space and degrees of angulation of impacted mandibular third molars as increasing age in sample 18 to 26 years. Furthermore, this study found no significant difference in impacted third molar positions between BMI categories. A study by Akinbami and Didia 9 suggested that BMI cannot predict the occurrence of mandibular third molar impaction. Instead, they found that impaction is predicted by the available retromolar space and mandibular length, which aligns with our findings.

Counting chest or abdomen movement through visual assessment is one method used to measure respiratory rate, 28 29 and this was the approach employed in this study. The findings of this study revealed that a higher respiratory rate was significantly correlated with shorter surgical time. Patients with an elevated preoperative respiratory rate may unconsciously prompt surgeons to respond to signs of patient discomfort or anxiety. Consequently, surgeons may strive to work more efficiently, aiming to minimize the overall duration of the procedure—a potential instinct to mitigate any negative impacts on the patient. However, it is crucial to validate this presumption in future studies, as the reliability of manually counting respiratory rate through visual assessments has been a subject of inquiry. 30

This study found no significant correlation between surgical time or surgical technique and all the positions of third molars. Several studies such as by Obimakinde et al, 31 discovered a significant contribution of third molar angulation to increased operation time. Similarly, Bello et al 32 found increased surgical time associated with distoangular and horizontal angulation. The disparity in findings in this study and the previous studies may be attributed to the different measurements used, where previous study used Winter classification for impaction angle, while this study used linear measurement. A little difference in angulation degree may cause different angulation classifications.

Numerous indices have been proposed to predict surgical difficulty, but their translation into clinical settings remains limited. 33 The ongoing exploration of variables crucial to surgical difficulty is imperative, allowing for the refinement of existing indices and the development of new ones with greater applicability in routine clinical practice. This continuous research holds the potential to enhance decision-making processes and improve outcomes in impacted third molar surgery. Regular updates and adjustments to predictive models based on these variables will contribute to a more precise and efficient assessment of surgical difficulty in oral and maxillofacial surgery.

## Conclusion

This study found that males have greater third molar eruption space than females, and maxillary third molars have a greater distance to the occlusal plane compared with mandibular third molars. The importance of vital signs as contributing factors to surgical difficulty is highlighted, emphasizing their relevance in clinical practice. Future research should explore additional variables influencing surgical difficulty to refine existing indices for broader clinical applicability.
